# Cold Feet

**Published:** 1883-02

**Authors:** 


### COLD FEET

Are the avenues to death of multitudes every year: it is a sign of imperfect circulation, of want of vigor of constitution. No one can be well, whose feet are habitually cold. When the blood is equally distributed to every part of the body, there is general good health. If there be less blood at any one point than is natural, there is coldness; and not only so, there must be more than is natural at some other part of the system, and there is fever, that is, unnatural heat or oppression. In the case of cold feet, the amount of blood wanting there, collects at some other part of the body which happens to be the weakest, to be the least able to throw up a barricade against the in-rushing enemy. Hence, when the lungs are weakest, the extra blood gathers there in the shape of a common cold, or spitting blood. Clergymen, other public speakers, and singers, by improper exposures often render the throat the weakest part; to such, cold feet gives hoarseness or a raw burning feeling, most felt at the little hollow at the bottom of the neck. To others, again, whose bowels are weak through over-eating, or drinking spirituous liquors, cold feet give various degrees of derangement, from common looseness up to diarrhœas or dysentery; and so we might go through the whole body, but for the present, this is sufficient for illustration.

*If you are well, let yourself alone.* This is our favorite motto. But to those whose feet are inclined to be cold we suggest,

As soon as you get up in the morning put both feet at once in a basin of cold water, so as to come half way to the ankles; keep them in half a minute in winter, a minute or two in summer, rubbing them both vigorously, wipe dry, and hold to the fire, if convenient, in cold weather, until every part of the foot feels as dry as your hand, then put on your socks or stockings.

On going to bed at night, draw off your stockings and hold the feet to the fire for ten or fifteen minutes until perfectly dry, and get right into bed. This is a most pleasant operation, and fully repays for the trouble of it. No one can sleep well or refreshingly with cold feet. All Indians and hunters sleep with their feet to the fire.

Never step from your bed with the naked feet on an uncarpeted floor. I have known it to be the exciting cause of months of illness.

Wear woollen, cotton or silk stockings, whichever keeps your feet most comfortable; do not let the experience of another be your guide, for different persons require different articles; what is good for a person whose feet are naturally damp, cannot be good for one whose feet are always dry. The donkey who had his bag of salt lightened by swimming a river, advised his companion who was loaded down with a sack of wool to do the same, and having no more sense than a man or woman, he plunged in, and in a moment the wool absorbed the water, increased the burden many fold, and bore him to the bottom.

